# Reliability of Glutamate Quantification in Human Nucleus Accumbens Using Proton Magnetic Resonance Spectroscopy at a 70-cm Wide-Bore Clinical 3T MRI System

**DOI:** 10.3389/fnins.2017.00686

**Published:** 2017-12-05

**Authors:** Xi-Long Liu, Long Li, Jian-Neng Li, Jia-Hui Rong, Bo Liu, Ze-Xuan Hu

**Affiliations:** Department of Radiology, Guangdong Provincial Corps Hospital of Chinese People's Armed Police Forces, Guangzhou Medical University, Guangzhou, China

**Keywords:** nucleus accumbens, glutamate, magnetic resonance spectroscopy, quantification, reliability

## Abstract

The human nucleus accumbens is a challenging region to study using proton magnetic resonance spectroscopy (^1^H-MRS) on a 70-cm wide-bore clinical 3T MRI system. The aim of this study was to investigate the reliability for quantitative measurement of glutamate concentration in the nucleus accumbens using a 70-cm wide-bore clinical 3T MRI. ^1^H-MRS of the nucleus accumbens was acquired using the Point-Resolved Spectroscopic Sequence (PRESS) with echo time of 40 ms from 10 healthy volunteers (5 female; age range: 18–30 years) on two separate visits (a baseline, and 1-month time point). The Java-based Magnetic Resonance User Interface (jMRUI) software package was used to quantitatively measure the absolute metabolite concentrations. The test-retest reliability and reproducibility were assessed using intraclass correlations coefficients (ICC), and coefficients of variation (CV). Glutamate concentrations were similar across visits (*P* = 0.832). Reproducibility measures for all metabolites were good with CV ranging from 7.8 to 14.0%. The ICC values of all metabolites for the intra-class measures were excellent (ICC > 0.8), except that the reliability for Glx (glutamate + glutamine) was good (ICC = 0.768). Pearson correlations for all metabolites were all highly significant (*r* = 0.636–0.788, *P* < 0.05). In conclusion, the short-echo-time PRESS can reliably obtain high quality glutamate spectrum from a ~3.4 cm^3^ voxel of the nucleus accumbens using a 70-cm wide-bore clinical 3T MRI.

## Introduction

The human nucleus accumbens, which considered to be a major part of the ventral striatum, is a limbic–motor interface involved in several cognitive, emotional, and psychomotor functions (Mavridis et al., 2011; Floresco, 2015). The nucleus accumbens belongs to the subcortical telencephalic structures that play a vital role in motivation. Dysfunction of nucleus accumbens is known to be associated with several neuropsychiatric disorders, including Parkinson's disease, Alzheimer's disease, depression, schizophrenia, obsessive-compulsive disorder, anxiety, and substance use disorders (Neto et al., 2008; Scofield et al., 2016). Proton magnetic resonance spectroscopy (^1^H-MRS) is a noninvasive technique used to elucidate neurochemical alterations in disease state and their reversal with therapies has great interest for studying the nucleus accumbens. As a supplemental mode to conventional structural magnetic resonance imaging (MRI), spectroscopy can help to distinguish by structural MRI indistinguishable pathologies and is sensitive to early cell metabolic changes in brain diseases (Bednarík et al., 2015). However, the role of nucleus accumbens spectroscopy in clinical research has been limited by the challenges associated with acquiring consistently high-quality MRS data from this small voxel region, especially high-quality glutamate spectroscopy. Additionally, metabolites alterations have been begun probing in illness state and treatment in psychiatric studies. These populations may be accepted a significant number of MRS scans. Therefore, it is important to establish such reliability with consistent testing times.

Glutamate is a principal excitatory neurotransmitter in the central nervous system. Previous research has demonstrated that glutamate can be noninvasively measured in different brain regions using ^1^H-MRS. For glutamate spectroscopy acquisition approaches, it cloud be divided into three categories: one-dimensional ^1^H MRS, and two-dimensional ^1^H MRS, and one-dimensional ^13^C MRS (Ramadan et al., 2013). There have been conflicting reports in the literature about the approaches used one-dimensional ^1^H MRS to detect glutamate in humans, such as TE-averaged PRESS (Hurd et al., 2004), optimal TE (Schubert et al., 2004; Jang et al., 2005, Mullins et al., 2008; Yang et al., 2008; Lally et al., 2016) and glutamate amine group-water chemical exchange saturation transfer (Cai et al., 2012, 2013), etc. However, most research was focus on the cingulate and parietal region, and used a standard volume (typically 8 mL) for spectroscopy acquisitions (Hurd et al., 2004; Yang et al., 2008). Furthermore, metabolite quantitative methods are most often expressed as ratios (relative quantification) rather than as absolute concentrations. Despite the great advantage that it is very easy to implement, it could easy lead to misinterpretation of spectral data and to erroneous metabolite values (Jansen et al., 2006). Absolute quantification is available, has an added value, and can improve the diagnostic utility of MRS (Jansen et al., 2006; Helms, 2008; Mandal, 2012). It can be obtained benefit from unambiguous interpretation and less prone to error. At present, the absolute quantification measurement methods *in vivo* using ^1^H MRS include external reference method, replace-and-match method, and water signal reference method (Jansen et al., 2006; Helms, 2008). The implementation of absolute quantification has been greatly facilitated in clinical routine.

The goals of this study were to investigate test-retest reliability for obtaining high-quality spectra, and to quantify the glutamate concentration in the human nucleus accumbens using single-voxel MR spectroscopy employing a clinical 3T scanner with 70-cm wide bore.

## Materials and methods

### Subjects

Ten right-handed healthy volunteers (5 male, 5 female, mean age: 24.6 ± 3.6 years, age range: 18–30 years) who were non-smokers, and without a previous history of neuropsychiatric or other diseases known to affect brain function or a contraindication to the MRI examination, were recruited using advertisements in the local press between March and October in 2016. All subjects signed and dated an informed consent form in accordance with procedures approved by the local Institutional Review Board. Each subject underwent MRI scanning twice including a baseline (Visit 1) and 1-month time point (Visit 2). Each subject was scanned at approximately the same time of day each time. Data acquisition and post-processing were performed by a qualified technologist.

### Data acquisition

**Figure 1:**
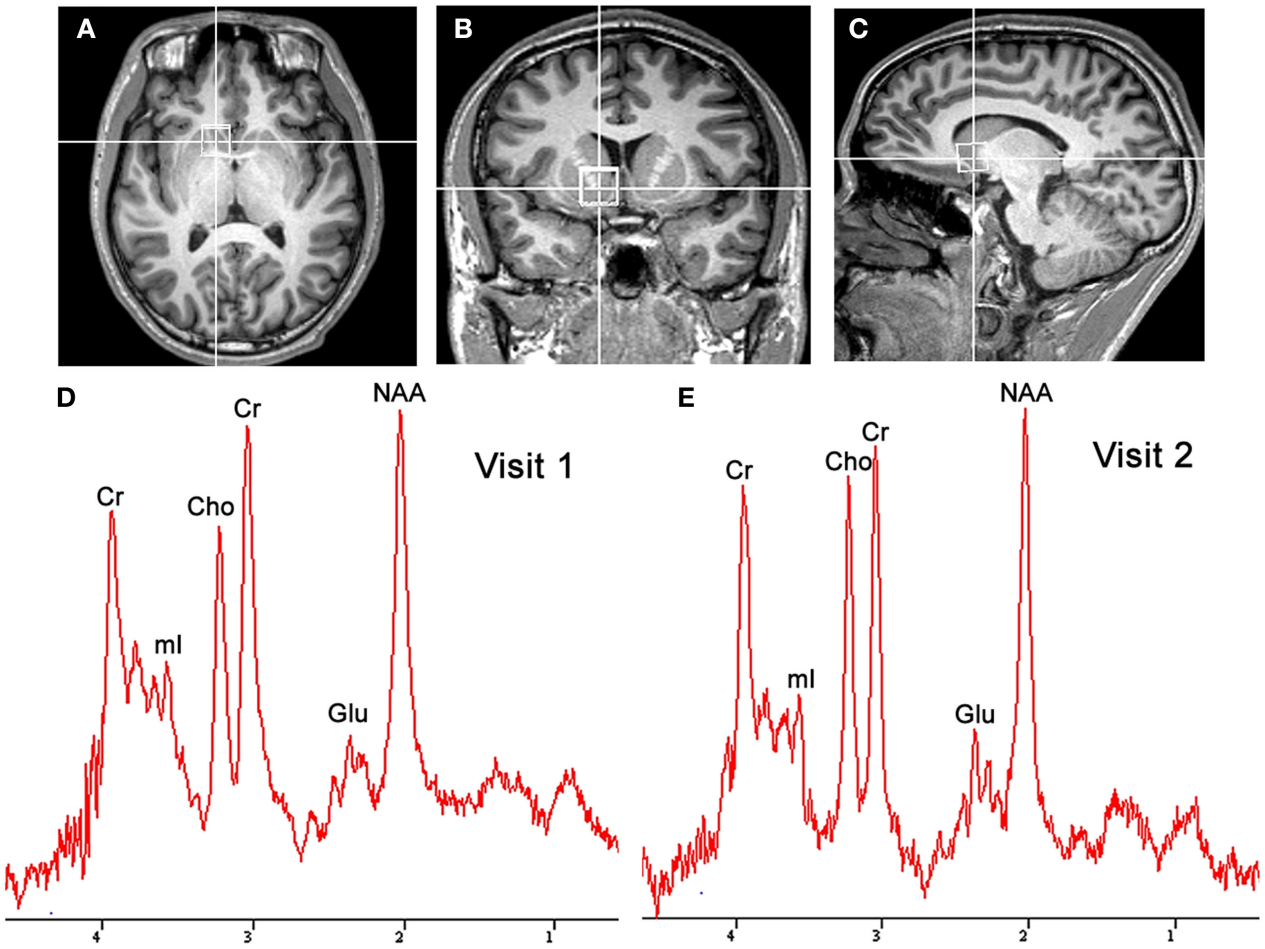
Localized images and representative MR spectra of the nucleus accumbens in the same participant. Localized images of the nucleus accumbens in the axial plane **(A)**, coronal plane **(B)**, and sagittal plane **(C)** are shown in the listed above. Representative spectra **(D,E)** from a 1.5 × 1.5 × 1.5 cm^3^ voxel in the nucleus accumbens for successive scans in the same participant are shown in the listed below. Cho, choline; Cr, creatine; Glu, glutamate; mI, myo-inositol; NAA, N-acetylaspartate.

Both MRI and ^1^H-MRS were performed using a Siemens MAGNETOM Skyra 3.0T MR scanner (Siemens Healthineers, Erlangen, Germany) equipped with the 20-channel phased-array joint head and neck coil. Various foam pads and a forehead-restraining strap were used to ensure head fixation during the scanning procedure. Subjects were positioned to lie comfortably in the coil, and asked to remain motionless during the procedure. High-resolution T_1_-weighted anatomic images of the whole brain were acquired by using a three-dimensional fast gradient-echo pulse sequence: TR = 2,300 ms, TE = 2.98 ms, TI = 900 ms, FOV = 256 × 256 mm, slice thickness = 1.0 mm, flip angle = 9°, TA = 5 min 12 s. Images from three orthogonal planes were revealed by using multiplanar reconstruction for localizing the spectroscopic volumes of interest (VOI: 15 × 15 × 15 mm). The VOI was placed to cover the most ventral part of the striatum in the coronal and sagittal slices of the reconstructed images of the ventral corner of the lateral ventricle as a topographic marker point (Figures 1A–C) (Bauer et al., 2013). To insure for consistency in voxel positioning between test-retest scanning, a screenshot of voxel placement in sagittal, axial and coronal section on the first scanning was created, and used it to guide the second scanning session.

All MRS data were acquired by using single-voxel localization. Spectral data were obtained by using a J-resolved Point-Resolved Spectroscopic Sequence (PRESS) with an echo time (TE) of 40 ms (Mullins et al., 2008). Voxel-speific first shimming was performed automatically using an automated shimming routine. Afterwards, to achieve unsuppressed water line width in the range of 7 to 10 Hz, the interactive shim function had further been refined manually. These spectra were also acquired with a TR of 2,000 ms, with 128 averages, giving a total scan time of over 4.5 min. The raw data from each acquisition were composed of 1,024 points at a bandwidth of 1,200 Hz. The total examination time was approximately 10 min. For quality control, a phantom in which the concentration of 50 mM creatine in a buffered salt solution (pH 7.2) at a temperature of 37°C, was measured in voxels of the same size at the center of metabolite phantoms during each MRS session using the same protocol.

### MRS data analysis

MRS data analysis was performed by using the Java-based Magnetic Resonance User Interface (jMRUI) v. 5.0 (http://www.mrui.uab.es/mrui/). The jMRUI software package enables the time-domain analysis of *in vivo* MRS data in two stages: preprocessing and quantization. Preprocessing techniques included eddy current compensation, Hankel–Lanczos singular value decomposition filtering (HLSVD), offset correction, zero filling, apodization, phase correction, and baseline correction (Mandal, 2012). Before fitting, spectra were preprocessed automatically by eddy current correction and phase coherent frequency shift correction, resulting to an improvement of the signal-to-noise ratio (SNR). Pre-processing requires user interaction using the HLSVD filter largely to suppress residual water molecules, and use of the Cadzow function to filter the signal. The quantities of interest were calculated with the Advanced Method for Accurate, Robust, and Efficient Spectral fitting (AMARES) (Vanhamme et al., 1997). AMARES fitting ensures the incorporation of more prior knowledge on the spectral parameters to increase efficiency, overall accuracy, and, convergence rates and can also be extended to fit echo signals. AMARES is preprogrammed to switch between the Lorentizian, Gaussian, and Voigt models, and can be used for fitting spin echoes in addition to free induction decay (Naressi et al., 2001; Mandal, 2012). Spectral fitting used Lorentzian line shapes. In order to improve the quantification process, this method depends on a prior knowledge entered by the user of the sought resonance peaks. A range of peak line width for the variation was also entered. These were allowed to vary between 2 and 14 Hz. The same prior knowledge to estimate peaks set at the following positions: 2.02 parts per million (ppm) and 3.9 line width [LW(Hz)] for N-acetylaspartate, 2.35 ppm and 4.9 LW for glutamate, 3.01 ppm and 4.9 LW for creatine, 3.2 ppm and 4.9 LW for choline (Cuellar-Baena et al., 2011; Scott et al., 2016). MRS data processed using AMARES in the jMRUI software package provided information about the estimated components, including frequencies, damping, amplitudes, and phases. Cramer–Rao lower bounds (CRLBs) were used as a measure of the accuracy of a calculation of the amplitude of a certain component. Only metabolite concentrations with CRLBs below 20% were accepted and used in subsequent analyses. The concentrations of metabolites were calculated according to as described in detail previously (Helms, 2008), as follows

(1) $$C=\frac{C_{\text{ext}}}{{\text{S}}_{\text{ext}}\text{/}({\text{V}}_{\text{ext}}{\text{R}}_{\text{ext}})}\frac{\text{S}}{\left(\text{VR}\right)}$$

where *C* stands for concentration, S for signal intensity, V for size of the VOI, R for the receiver gain, and ext for external reference.

Numerous effects should be taken into account, when performing MRS on a phantom, and getting absolute concentration estimates requires a calibration. First, the measurement on phantom is generally performed at a lower temperature (Temp) than the human body temperature, so yielding a polarization increased by 310 K/Temp. The relaxation times, T_1_ and T_2_, which are normally somewhat longer *in vitro* than *in vivo*, will also be impacted by the temperature. Scaling the reference signal to match the conditions *in vivo* should be considered. Additionally, a mismatch of coil impedance along with the associated scaling and reflection losses, due to the load of the phantom is small, as described in detail previously (Helms, 2008).

(2) $${\text{S}}_{\text{ext}}^{vivo}\;=\;{\text{S}}_{\text{ext}}\frac{\text{Temp}}{310\text{K}}\frac{\text{exp}(-{{\text{TE/T}}_{\text{2}}}^{vivo})}{\text{exp}(-{{\text{TE/T}}_{\text{2}}}^{vitro})}\frac{\left[1-\text{exp}({{-\text{TE/T}}_{\text{1}}}^{vivo})\right]}{\left[1-\text{exp}({{-\text{TE/T}}_{\text{1}}}^{vitro})\right]}$$

where S stands for signal intensity, Temp for temperature of the phantom, and ext for external reference. The results for the metabolites of interest were then corrected for partial volume and relaxation effects as an average of the metabolites of interest reported in the literature. Briefly, for N-acetylaspartate, T_1_ = 1.47 s and T_2_ = 247 ms; for choline, T_1_ = 1.30 s and T_2_ = 207 ms; for creatine, T_1_ = 1.46 s and T_2_ = 152 ms; for glutamate, T_1_ = 1.27 s and T_2_ = 199 ms (Mlynárik et al., 2001; Mullins et al., 2008).

### Statistical analysis

SPSS 13.0 (Chicago, USA) was used for the data analysis. The MRS data were screened according to the residual water peak, baseline, CRLBs, and additional criteria described above before analysis. Only metabolite concentrations with CRLBs below 20% were accepted. We first assessed whether there was a main effect of gender or scanning time, or an interaction between those two factors using a two factor, repeated measured ANOVA (sex, visit). Then, we assessed differences in glutamate concentration across time in the nucleus accumbens using one-factor repeated measures ANOVA. The intraclass correlation coefficient (ICC, 2,1) was used to assess the reliability by comparing metabolites from the first and second scan (Visit 1 vs. Visit 2). ICC is considered the best assessment for test-retest reliability. An ICC value of <0.6, 0.6–0.7, 0.7–0.8, and ≥0.8 represents poor, fair, good, and excellent reliability, respectively (Lally et al., 2016). A coefficient of variation (CV) was also computed across days for each metabolite in the nucleus accumbens. Data is reported as mean ± standard deviation. Additionally, Pearson correlations were calculated for two scans to test reliability for each metabolite. Results were classified as significant if *P* < 0.05.

## Results

**Figure 2:**
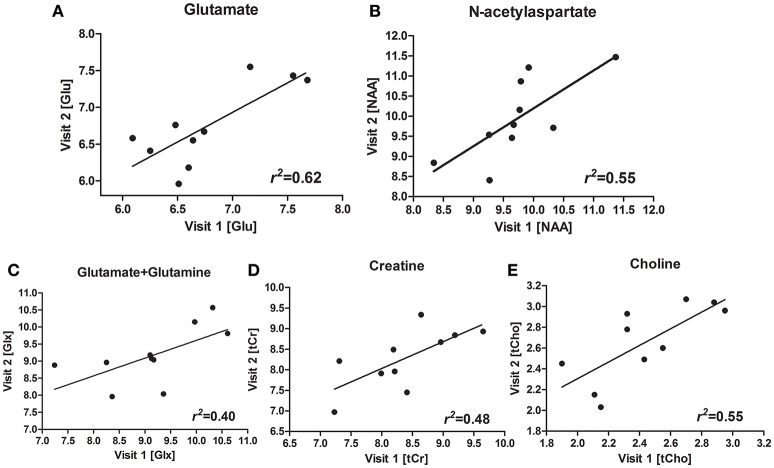
Scatterplots and regression line for glutamate (Glu) **(A)**, N-acetylaspartate (NAA) **(B)**, glutamate + glutamine (Glx) **(C)**, creatine (Cr) **(D)**, choline (Cho) **(E)**, for the two visits.

Figures 1D,E shows a representative spectrum in the nucleus accumbens from visit 1 and visit 2 within an individual. The spectral data of metabolites (N-acetylaspartate, glutamate, Glx (glutamate + glutamine), creatine, and choline) were acquired successfully in all participants with CRLB ≤ 20% using the conventional PRESS in the 10 volunteers. Table 1 summarizes the mean metabolites value and standard deviation for visits. Glutamate concentrations were similar across visits (*P* = 0.832). The ICC values of all metabolites for the intra-class measures were excellent (ICC > 0.8), except that the reliability for Glx was good (ICC = 0.768, Table 1). Pearson correlations for all metabolites were all highly significant as showed in Table 1 and Figures 2A–E.

**Table 1:** Metabolite concentrations means (standard deviations) in the nucleus accumbens from visit number, intraclass correlation coefficient (ICC), coefficient of variation (CV, %) and Pearson correlation between visit number.

	**Visit NO**.	**Mean(SD), mM**	**CV, (%)**	**ICC(2,1)**	**Pearson *r***	***P***
NAA	1	9.736 (0.779)	8.0	0.836	0.739	0.015
	2	9.946 (0.994)	10.0			
Glu	1	6.770 (0.529)	7.8	0.881	0.788	0.007
	2	6.746 (0.540)	8.0			
Glx	1	9.151 (1.016)	11.1	0.768	0.636	0.048
	2	9.167 (0.832)	9.1			
tCr	1	8.378 (0.772)	9.2	0.820	0.696	0.025
	2	8.277 (0.723)	8.7			
tCho	1	2.431 (0.341)	14.0	0.850	0.742	0.014
	2	2.650 (0.368)	13.9			

*ICC, intraclass correlation coefficient; CV, coefficient of variation; NAA, N-acetylaspartate; Glu, glutamate; Glx, glutamate + glutamine; tCr, total creatine; tCho, total choline*.

No main effects were observed for sex (*P* = 0.42) and sex-by-visit interaction (*P* = 0.73). We also conducted a one-factor repeater measures ANOVA to test the stability between visit 1 and visit 2, and showed sufficient test-retest reliability. All metabolites showed no significant change from visit 1 to visit 2 (all *P* > 0.10), except for choline, *F*_(1, 9)_ = 6.596, *P* = 0.03.

## Discussion

This study demonstrated the reliability of glutamate measures in the nucleus accumbens, a region particularly challenging for ^1^H-MRS. The results presented in this report show that the short-echo-time PRESS can be used to measure glutamate concentrations in the nucleus accumbens, and glutamate concentrations remained consistent over the two testing scans across 1 month. We have shown here that ^1^H-MRS provided clinically acceptable test-retest reliability in quantifying glutamate concentrations within an individual over time in a traditionally challenging region of the brain, the nucleus accumbens.

Using a smaller VOI (3.4 cm^3^) compared with other reproducibility and reliability studies, our result showed excellent reliability for detecting metabolite concentrations in the nucleus accumbens using ^1^H-MRS performed with a 3.0T wide-bore clinical scanner. Similar results were reported from previous spectra research. Schubert et al. (2004) reported that *in vivo* data acquired from a 2.5 × 4 × 2 cm^3^ voxel located in the anterior cingulated cortex (ACC) and a 2 × 3 × 2 cm^3^ voxel located in hippocampus had concentrations of glutamate and glutamine in the ACC of 11.7 ± 1.2 mM and 2.5 ± 0.8 mM, respectively, while glutamate and glutamine were found to be 10.9 ± 1.4 and 2.2 ± 0.8 mM in the hippocampus, respectively. Jang et al. (2005) reported results that *in vivo* spectra acquired from in ACC (voxel size: 2 × 2 × 1 cm^3^), where a glutamate concentration of 10.51 ± 1.6 mM, and a glutamine concentration of 4.70 ± 1.00 mM were found. Similarly, Mullins et al. (2008) reported a glutamate concentration of 13.14 mM and a glutamine concentration of 2.35 mM in the ACC (voxel size: 2 × 2 × 3 cm^3^). Although it is difficult to directly compare the results of this study with those of previous studies—owing to differences in data acquisition techniques, voxel location, and quantification methodology—the results presented suggest that reproducible and reliability measurements of the concentration of glutamate can be obtained from single-voxel spectroscopic acquisitions. However, the measurement of glutamate is complicated by its complex J-coupled spectrum and overlapping peaks from other metabolites, primarily glutamine (Schubert et al., 2004; Yang et al., 2008). The C4 proton resonance multiplets around 2.35 ppm for glutamate and 2.45 ppm for glutamine commonly show various degrees of spectral overlap at different field strengths. The two resonances in the region of 2.3–2.5 ppm completely collapse and are usually termed as Glx at low field strength (e.g., 1.5T), and mainly exists the two outer wings overlap at mid-field strengths (e.g., 3 or 4T), and separated at higher field strengths (e.g., 7 and 9.4T) (Yang et al., 2008). Hence, there could be increased challenges to curve fitting at low- and mid-field strengths. In our MRS data analysis, we fitted the glutamate spectral peak as a single peak at 2.35 ppm. Although the glutamate spectral peak was a multiplet proton resonance at 2.35 ppm, this method could reduce the impact of outer wings from the spectral overlap, leading to the underestimation of the quantitative results of glutamate. Additionally, various experimental conditions could impart imperfections to the MRS data. However, with the improvements in equipment and technology, such as scanner hardware, MRS acquisition methodology, processing and analysis, it is likely that the glutamate quantity of clinical MRS will markedly improve.

In the present study such a relatively small VOI is used for location because the volume of nucleus accumbens is relatively small, and the nucleus accumbens presented in the shape of a convexo-convex round with a flat dorsum and located symmetrically in the front of the anterior commissures in both cerebral hemispheres. Previous morphological studies had difficulty establishing the rostral end of the nucleus accumbens relative to the caudate nucleus and putamen, and had not a distinct signal intensity imaging (Neto et al., 2008; Mavridis et al., 2011). Neto et al. (2008) reported that the nucleus accumbens dimensions were 10.5 × 14.5 × 7.0 mm (length, width, and height, respectively). In our study, the voxel was 15 × 15 × 15 mm, able to cover the whole nucleus accumbens. In addition to the ventral striatum, white matter belonging to the internal capsule was also included in our VOI. Therefore, the result will be overestimated. But we reasoned that a smaller voxel would increase anatomical precision and improve shimming, while the lower signal could be improved with additional signal averages.

In this study the metabolites in the nucleus accumbens are measured with standard single-voxel ^1^H-MRS PRESS methods at a 3T MRI system and without sophisticated editing techniques. Although other reports have shown better reproducibility for glutamate, this could be a result of SNR and different fitting techniques. Compared with other methods, optimal TE methods are easy acquisition and processing on clinical 3T MRI system, and the resulting spectra can be analyzed using the scanner or commercially available software (Mullins et al., 2008; Ramadan et al., 2013). Moreover, data acquisition using PRESS doubles the signal compared with stimulated echo acquisition mode (STEAM), leading to increased SNR. However, PRESS cannot achieve the very short echo time that STEAM can (Wijtenburg and Knight-Scott, 2011). Despite this weakness, the technique has successfully been employed to acquire high-quality spectral imaging on a clinical wide-bore MRI system at 3T.

In the present study short-echo-time ^1^H-MRS techniques simplify the task of spectral modeling, but could suffer from increased spectral overlap and increased challenges to curve fitting and quantification. For quantification, we used the external reference method, which involves a vial with a known reference solution and relaxation properties positioned near or inside the radiofrequency coil. Generally speaking, the signal spectrum for the reference solution is a single, narrow line with high SNR. However, because this reference signal comes from a different spatial region, the reference signal will be affected by B1 inhomogeneities in the radiofrequency field (Jansen et al., 2006). The external vial could also introduce substantial distortions of the constant magnetic induction field homogeneity, which would complicate shimming and water suppression. Additionally, the T_1_ and T_2_ relaxation times are not always the same *in vitro* and *in vivo*, and different resonances in the same metabolite can have different relaxation times. For example, the external phantom is likely to have a higher signal at the same TE due to a longer T_2_ in solution. In all of these cases, the signal intensities of each resonance have to be properly corrected for T_2_ relaxation (e.g., Equation 2).

Magnet homogeneity is a key parameter in the capability of an MRI system especially in MRS. The first wide-bore systems appeared in the market around 2004. At that time, the poorer magnet homogeneity and gradient performance were pointed out (Sobol, 2012). Compared with a traditional 60-cm MRI system, a larger-bore system has reduced B_0_ magnet homogeneity. At high field strengths, the wavelength of the radiofrequency (RF) approaches the dimensions of the human anatomy. This can create destructive excitation field interference and non-uniform signal intensities in the imaging volume. To solve this problem, MRI manufacturers use various RF subsystems, including the total image matrix from Siemens, geometry-embracing method from GE, and dStream from Philips, to improve the magnet homogeneity (Sobol, 2012). The results of clinical practice have showed that a 70-cm wide-bore magnet system has realized the perfect combination of the high-quality imaging with the patient comfort (Kim et al., 2015; Tarnoki et al., 2015; Saito et al., 2016). In this study the high resolution spectra of metabolites included glutamate are acquired reliably at a 70-cm wide-bore magnet system.

This is one limitation of the present study that the sample size is very small for test-retest reliability statistics. One promising direction of getting a large scale neuroimaging MRS data could be a consortium like functional MRI (Zuo et al., 2014).

## Conclusion

In sum, our study is the first which involved that high quality glutamate spectrum can reliability be obtained from a ~3.4 cm^3^ nucleus accumbens voxel using a 70-cm wide-bore clinical 3T MRI system with the short-echo-time PRESS. Studies using ^1^H-MRS have reported differences in glutamatergic neurotransmitter concentrations in nucleus accumbens between healthy and clinical populations such as alcohol-dependent or opioid-dependent (Bauer et al., 2013; Liu et al., 2017). Reliable quantification of glutamate using ^1^H-MRS with a wide-bore clinical 3T MRI system would be beneficial for clinical studies relating to glutamate in the human nucleus accumbens.

## Ethics statement

This study was carried out in accordance with the recommendations of the board of ethics committee based on the regulation of China Ministry of Health “Procedures for Ethical Review for Biomedical research involving humans subjects,” the WMA Declaration of Helsinki, the CIOMS International Ethical Guidelines for Biomedical Research Involving Human Subjects, and the WHO Operational Guidelines for Ethics Committees That Review Biomedical Research. The protocol was approved by the Medical Ethics Committee of Guangzhou Medical University.

## Author contributions

LL and X-LL designed the work. X-LL, J-NL, J-HR, BL, and Z-XH did the acquisition, analysis, or interpretation of data for the work. All the authors drafted the work and revised it critically for important intellectual content approved the final version to be published.

### Conflict of interest statement

The authors declare that the research was conducted in the absence of any commercial or financial relationships that could be construed as a potential conflict of interest.
